# Physicochemical Properties of Chitosan from Green Mussel Shells (*Perna viridis*): A Comparative Study

**DOI:** 10.3390/polym15132816

**Published:** 2023-06-26

**Authors:** Pimonpan Kaewprachu, Chalalai Jaisan

**Affiliations:** College of Maritime Studies and Management, Chiang Mai University, Samut Sakhon 74000, Thailand; chalalai.jai@cmu.ac.th

**Keywords:** chitosan, biopolymer films, by-product, green mussel shells, physicochemical properties

## Abstract

Green mussel shells (*Perna viridis*) are generated in huge amounts and discarded as waste materials. Such waste may be used to produce biopolymer materials such as chitosan. The physicochemical properties of chitosan prepared from different sizes of green mussel shells (small size (CHS): ≤5.00 cm in length and big size (CHB): >5.01 cm in length) were characterized and compared with commercial chitosan (CH). Furthermore, the mechanical and physicochemical properties of the blended films were also investigated. The results of the physicochemical properties showed that CHS and CHB were quite different from CH. The degree of deacetylation of CHS, CHB, and CH was found to be 32.71%, 52.56%, and 70.42%, respectively (*p* < 0.05). The water- and fat-binding capacities of CH were higher than those of CHS and CHB. Structural differences between CHS, CHB, and CH were studied using Fourier transform infrared spectroscopy (FTIR) and X-ray powder diffraction (XRD). Significant increases in thickness, water vapor permeability, and strength of the blended films were found when the extracted chitosan was added (*p* < 0.05). However, further study is needed to improve the chitosan extraction process, which can enhance the physicochemical properties of the obtained chitosan and be widely used in many industries.

## 1. Introduction

Green mussel (*Perna viridis*) is an economic seafood product and is commonly consumed in Thailand. In 2021, the highest production quantity of shellfish from culture farms in Thailand was that of green mussels (52,067.29 tons, or 52.64% of the total production quantity of shellfish), followed by bloody cockle (33,526.27 tons, or 33.90%) and oyster (13,317.41 tons, or 13.46%) [1]. The seafood processing industry in Thailand, especially in Laem Yai, Samut Songkhram province, generally uses green mussels to produce pickled and dried mussels. After processing, the shells are generated in huge amounts; around 70% of the total weight of the whole shell is wasted material. As a result, many thousands of tons of mussel shells are left over from the seafood processing industry per year, disposed of in landfills, and discarded in public areas, which causes environmental and health problems. It has been reported that the green mussel shells contain chitin compounds in a range of 14–35% [2,3], which is suitable for use as a starting material to produce biomaterials such as chitosan. Thus, this is an alternative way to add value to these by-products. It also reduces the accumulation of waste from green mussel shells that causes environmental pollution.

Chitosan is a polysaccharide and a biopolymer derivative of chitin that is obtained from the deacetylation process. It is a linear polymer comprising β-(1,4)-2-acetamido-2-deoxy-d-glucose and β-(1,4)-2-anaino-2-deoxy-d-glucose units [4]. The main sources of chitosan are the exoskeletons of crustaceans, the cell walls of fungi, some mollusks, and insects [5,6]. These starting materials consist of other compounds, such as protein, lipid, pigment, and calcium carbonate, that should be removed before the deacetylation process. The chemical extraction process of chitosan generally includes deproteinization, demineralization, decolorization (if any), and deacetylation [6]. Usually, chitin is obtained after exposing the exoskeletons of invertebrates to acidic and alkali conditions to dissolve calcium carbonate and protein, respectively. Chitin can be converted to chitosan by exposing it to concentrated alkali solutions, such as 50% NaOH, at high temperature (above 100 °C). In addition, the physicochemical, biological, and functional properties of the obtained chitosan are influenced by the variation in species, nature, location, the initial compositions of the raw material, chitosan processing methods (such as deproteinization, demineralization, decolorization, and deacetylation), and their conditions (heating temperature and time, and alkali concentrations) [7,8]. At present, the production of chitosan extracted from crustacean by-products or insects has been studied in crab shell [2], grasshopper, silkworm chrysalis [5], mealworm cuticles [5,9], shrimp shell [10], mantis shrimp shell [11], squid pen [12], *Pinna bicolor* shell [13], and horse mussel [14].

Chitosan is the most favored biopolymer due to its biocompatibility, biodegradability, and non-toxicity. It is widely applied in various industries, such as food, cosmetics, pharmaceuticals, and biomedical [6]. It can also be produced as biopolymer composite films [11,15,16]. However, the production of chitosan extracted from green mussel shells and its applications, such as biopolymer films, are relatively unexplored. Therefore, the aim of this study was to extract chitosan from the different sizes of green mussel shells (small size: ≤5.00 cm in length and big size: >5.01 cm in length) and investigate their physicochemical properties. The development of chitosan films blended with extracted chitosan and the properties of the blended films were also investigated. The results of this study may be useful for introducing an alternative resource of polysaccharides for extracting chitosan, and the obtained chitosan could be applied in many industries.

## 2. Materials and Methods

### 2.1. Materials

Green mussel shells (*Perna viridis*) were obtained from Laem Yai district, Samut Songkhram province, Thailand. Commercial chitosan powder (CH, obtained from the king crab shell with a 92.50% degree of deacetylation), hydrochloric acid, and sodium hydroxide were purchased from Union Science Co., Ltd. (Chiang Mai, Thailand). All other chemicals were of analytical grade.

### 2.2. Sample Preparation

The green mussel shells were shipped to the laboratory of the College of Maritime Studies and Management, Chiang Mai University, Samut Sakhon, Thailand. In the laboratory, the green mussel shells were sorted into two sizes: small size (≤5.00 cm in length) and big size (>5.01 cm in length), using manual measurement with a digital vernier caliper. The shells were washed several times with running tap water and dried overnight in a tray dryer at 70 °C. The dried shell samples were collected and kept in a polyethylene zip-locked bag. The samples were stored at room temperature for further use. The shell samples were subjected to proximate analysis (moisture, ash, fat, and protein) and calcium carbonate analysis.

### 2.3. Extraction of Chitosan from Green Mussel Shells

The green mussel shells were soaked in 0.5 M HCl in a ratio of 1:15 (*w*/*v*) at room temperature for 6 h to remove all organic materials, washed with distilled water, and dried in a hot air oven at 70 °C overnight. Deproteinization of both sizes of shells was performed by treating the pretreatment shells with 1 M NaOH in a ratio of 1:20 (*w*/*v*) at 95 °C for 4 h. The deproteinized shells were washed with running tap water until they reached a neutral pH, and were dried at 70 °C overnight. Demineralization of both sizes of shells was performed by treating the deproteinized shells with 1 M HCl at a ratio of 1:20 (*w*/*v*) at room temperature for 48 h. The demineralized shells were washed with running tap water until they reached a neutral pH, and were dried at 70 °C overnight. The product obtained was “chitin”. The extraction of chitosan involves the deacetylation of extracted chitin, as described in the report by Alishahi et al. [17] with slight modifications. The extracted chitin (20 g) was steeped in 400 mL of 50% NaOH for 24 h before being heated in an autoclave at 120 °C for 120 min. After that, the residue was washed with running tap water until it reached a neutral pH. The residue was filtered through a Bucher funnel and dried at 70 °C overnight. The obtained chitosan from small size and big size were referred to as “CHS” and “CHB”, respectively. The percentage yield of CHS and CHB was determined using Equation (1):

(1) $$\mathrm{CHS}\;\mathrm{or}\;\mathrm{CHB}\;\mathrm{yield}\left(\%\right)=\frac{\mathrm{Weight}\;\mathrm{of}\;\mathrm{CHS}\;\mathrm{or}\;\mathrm{CHB}\;(g)}{\mathrm{Weight}\;\mathrm{of}\;\mathrm{starting}\;\mathrm{shells}\;(g)}\times \;100$$

### 2.4. Characterization of Chitosan

#### 2.4.1. Chitosan Appearance and Color

A camera phone (Apple iPhone 8 Plus, Apple Inc., Cupertino, CA, USA) was used to photograph all samples.

The color of the CH, CHS, and CHB powders was analyzed using a colorimeter (ColorFlex EZ, HunterLab, Virginia, VA, USA). L*, a*, and b* values were used to express various color properties.

#### 2.4.2. Fourier Transform Infrared Spectroscopy (FTIR) Analysis and Determination of Degree of Deacetylation

The degree of deacetylation (DDA) of the CH, CHS, and CHB powder was estimated using Perkin Elmer FTIR Spectrometer Spectrum One (Perkin Elmer, Waltham, MA, USA) with a frequency range 4000–400 cm^−1^ with 64 scans at a resolution of 4 cm^−1^. The absorbance at A1650 and A3450 cm^−1^ indicate absolute heights of absorption bands of amide and hydroxyl groups, respectively. The DDA (%) was calculated using Equation (2) [7]:

(2) $$\mathrm{DDA}\left(\%\right)\;=100-[(\frac{A1650}{A3450})\times \frac{100}{1.33}]$$

#### 2.4.3. Solubility

The solubility of the CH, CHS, and CHB powders was measured by dissolving the sample (0.1 g) in 10 mL of 1% (*v*/*v*) acetic acid and stirring at room temperature for 1 h before subjecting the mixture to centrifugation at 3000× *g* for 10 min. The undissolved samples were separated and dried in an oven at 105 °C overnight and weighed. Then, the solubility of the CH, CHS, and CHB was calculated using Equation (3):

(3) $$\mathrm{Film}\;\mathrm{solubility}\left(\%\right)=\frac{\mathrm{Wi}-\mathrm{Wf}}{\mathrm{Wi}}\times \;100$$

where *Wi* and *Wf* are the initial weights of the CH, CHS, and CHB powders and the final dry weight of the undissolved CH, CHS, and CHB, respectively.

#### 2.4.4. Determination of Water-Binding Capacity

The water-binding capacity (WBC) of the CH, CHS, and CHB powders was estimated according to the method described by Kumari et al. [7]. A quantity of 10 mL of distilled water was added after the sample (0.5 g) had been weighed in a 50 mL centrifuge tube. After thoroughly blending, the mixture was left at room temperature for 30 min (remixing every 10 min). The mixture was then subjected to centrifugation for 25 min at 3200 rpm. The centrifuge tubes were weighed after the supernatant was discarded. The WBC value was determined using Equation (4):

(4) $$\mathrm{WBC}\left(\%\right)=\frac{\mathrm{Water}\;\mathrm{bound}\;(g)}{\mathrm{Initial}\;\mathrm{sample}\mathrm{weight}\;(g)}\times \;100$$

#### 2.4.5. Determination of Fat-Binding Capacity

The fat-binding capacity (FBC) of the CH, CHS, and CHB powders was estimated according to the method described by Kumari et al. [7]. A 50 mL centrifuge tube containing the sample (0.5 g) and 10 mL of soy oil was weighed initially. After thoroughly blending, the mixture was left at room temperature for 30 min (remixing every 10 min). The mixture was then centrifuged for 25 min at 3200 rpm. The centrifuge tubes were weighed after the supernatant was eliminated. The FBC value was determined using Equation (5):

(5) $$\mathrm{FBC}\left(\%\right)=\frac{\mathrm{Fat}\;\mathrm{bound}\;(g)}{\mathrm{Initial}\;\mathrm{sample}\mathrm{weight}\;(g)}\times \;100$$

#### 2.4.6. X-ray Diffraction (XRD) Analysis

The diffraction pattern of the CH, CHS, and CHB powders was detected using an X-ray diffractometer (Model D8 Discover, Bruker AXS, Germany) with Cu radiation (40 kV and 40 mA). The data were evaluated within the 2θ range of 5–80°.

### 2.5. Preparation of Extracted Chitosan/Commercial Chitosan Blended Films

The CH, CH/CHS, and CH/CHB blended films were prepared according to the method described by Rachtanapun et al. [18] with slight modifications. CH 1% (*w*/*v*) was mixed with 2% (*v*/*v*) acetic acid. The mixture was stirred at 65–70 °C for 30 min, and CHS or CHB (40%, *w*/*w* based on CH content) was added to the solution. After mixing for 30 min, 25% glycerol (*w*/*w*, based on CH content) was added and stirred for another 30 min. To remove the bubbles, the film-forming solution (FFS) of the CH (without the addition of extracted chitosan), CH/CHS, and CH/CHB blended FFS was placed in a 500 W ultrasonic cleaner (GT sonic-D27, GT Sonic, China) at 50 °C for 15 min. The FFS was poured on a silicone plate (50 × 50 mm) and dried in an oven at 40 °C for 24 h. The CH, CH/CHS, and CH/CHB blended films were continuously dried in a constant climate chamber (KMF 115, Binder GmbH, Tuttlingen, Germany) at 50 ± 5% relative humidity (RH) and 25 ± 0.5 °C for 24 h. The CH, CH/CHS, and CH/CHB blended films were preconditioned for 48 h at 50 ± 5% RH at 25 °C in a constant climate chamber (KMF 115, Binder GmbH, Tuttlingen, Germany) before testing.

### 2.6. Characterization of Chitosan Blended Films

#### 2.6.1. Film Thickness

A hand-held micrometer with 0.001 mm precision (Mitsutoyo Co., Kanagawa, Japan) was used to measure the thickness of the chitosan blended films. Ten samples were measured at six random locations all over each film.

#### 2.6.2. Mechanical Properties

A texture analyzer (TA.XT plus, Stable Micro Systems, Godalming, UK) coupled with a 50 kg load cell was used to assess the mechanical characteristics (tensile strength, TS and elongation at break, EAB) of the blended films. Each film (20 × 50 mm) was assessed using a 30 mm grip separation distance and a 10 mm/sec test speed.

#### 2.6.3. Films Appearance and Color

A camera phone (Apple iPhone 8 Plus, Apple Inc., Cupertino, CA, USA) was used to photograph the CH, CH/CHS, and CH/CHB films.

The color of the chitosan blended films was analyzed using a colorimeter (ColorFlex EZ, HunterLab, Virginia, VA, USA). L*, a*, and b* values were used to express various color properties.

#### 2.6.4. Water Vapor Permeability (WVP)

A modified ASTM E96-80 method [19] was used to evaluate the water vapor permeability (WVP) of the chitosan blended films. The aluminum cup, which had a 70 mm diameter circle inside that contained 10 g of silica gel at 0% RH, was covered with the film. The cup was kept at 75 ± 5% RH at 25 °C for 8 h in a constant climate chamber (KMF 115, Binder GmbH, Tuttlingen, Germany), and its weight was recorded hourly. The WVP values of the blended films were expressed as g m m^−2^ s^−1^ Pa^−1^.

#### 2.6.5. Fourier Transform Infrared (FTIR) Analysis

All films were used for FTIR analysis after being dried out for two weeks in a desiccator. A Perkin Elmer FTIR Spectrometer Spectrum One (Perkin Elmer, Waltham, MA, USA) operated at a resolution of 4 cm^−1^ was used to analyze the FTIR spectra of the chitosan blended films. The FTIR spectra were recorded in the range of 4000–650 cm^−1^ wavenumbers with 64 scans.

### 2.7. Statistical Analysis

All data were subjected to an analysis of variance (ANOVA). The differences between means were taken using Duncan’s Multiple Range Tests (*p* < 0.05). Statistical analysis was performed with SPSS software (SPSS for Windows version 16.0, SPSS Inc., Chicago, IL, USA).

## 3. Results and Discussion

### 3.1. Characterization of Green Mussel Shells

The composition of green mussel shells was very important as a basis for designing and evaluating the subsequent fractional procedure until chitosan was obtained because, to obtain a high quality of chitin, it was necessary to eliminate the two major components of green mussel shells (minerals and proteins). According to Table 1, the composition of small shells was 0.79% moisture, 42.63% ash, 0.03% fat, 5.13% protein, and 78.91% calcium carbonate, while that of big shells was 0.91% moisture, 92.90% ash, 0.03% fat, 4.51% protein, and 78.13% calcium carbonate. It can be seen that ash and calcium carbonate (CaCO_3_) are the dominant chemical constituents of both sizes of shells. The different sizes of shells showed differences in ash content, with the big size shell having a higher ash content than the small size shell. It can be concluded that the ash content of both sizes is positively correlated with the shell sizes. The quantity of ash present in both sizes of green mussel shells is relatively high when compared with the quantity of ash present in others, such as mealworm (Tenebrio molitor) cuticles (3.7%) [9] and house cricket (Brachytrupes portentosus) (2.4–5.8%) [20]. Furthermore, the amount of CaCO_3_ (78.13–78.91%) in green mussel shells in this study is relatively high when compared to the calcium carbonate content present in black tiger shrimp (50.12) [17], pink shrimp shells (42.26%), brown shrimp shells (48.97%), crab shells (66.58%), crayfish shells (63.94%), and squid pens (4.78%) [21]. It can be concluded that chemical compositions vary depending on nature, species, and location. This is consistent with Chakraborty et al. [22], who reported that the chemical composition of mussel shells was affected by shell length. The growth stages (size of the green mussel shell) were positively related to the accumulation of CaCO_3_ content [23]. Higher ash and calcium carbonate contents in starting materials could affect the extraction process and physicochemical properties of extracted chitosan. Alishahi et al. [17] reported that the amount of mineral in the starting materials could affect the period of demineralization; the more minerals, the longer the demineralization process needed. The chemical extraction processes, including demineralization, deproteinization, and/or decolorization, prior to the extraction of chitosan (the deacetylation process), are very important. Therefore, the use of effective extraction processes could provide chitin and chitosan having high purity.

### 3.2. Characterization of Chitosan Extracted from Green Mussel Shells

#### 3.2.1. Visual Appearance and Color Attributes of Chitosan Powders

**Figure 1:**
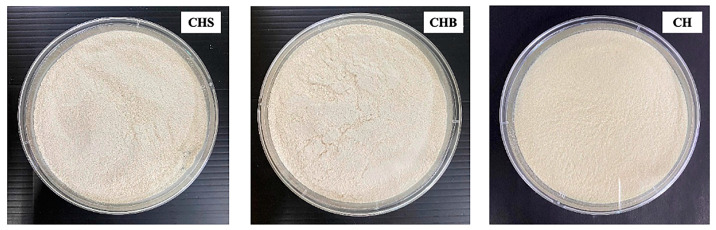
Visual appearance of chitosan obtained from small green mussel shells (CHS) and big green mussel shells (CHB) in comparison with commercial chitosan (CH).

The visual appearance of chitosan prepared from different sizes of green mussel shells (CHS and CHB) in comparison with commercial chitosan (CH) is shown in Figure 1. Visually, all chitosan powder samples were fine powder and homogenous. The CHS and CHB powders had a white color, while the CH powder had a light-yellow color. This result was related to the color values (L*, a*, and b*) of chitosan powders (Table 2). The extracted chitosan, both CHS and CHB, had a higher L* value (lightness), while CH had a higher b* value (yellowness) (*p* < 0.05). This might be related to the presence of β-1,4 linked 2-amino-2-deoxy-d-glucopyranose repeat units [24]. Furthermore, the color of extracted chitosan may also depend on the pigment present in the origin of the chitosan or extraction process parameters (time, temperature, concentration of NaOH) [11].

#### 3.2.2. The Percentage of Yield and Degree of Deacetylation (DDA)

The percent yield of chitosan extracted from both sizes of green mussel shells is shown in Table 2. The yield of chitosan extracted from small green mussel shells (CHS) was 0.225%, while the yield of chitosan extracted from big green mussel shells (CHB) was 0.079% (*p* < 0.05), which was calculated based on the weight of whole starting shells. The higher yield was observed in CHS. The lower yield of CHB might be due to the chitosan polymer’s depolymerization, the loss of sample weight from excessive removal of acetyl groups from the polymer during deacetylation, and the loss of chitosan particles during washing (to neutral pH) [4]. Furthermore, the yield of chitosan also depends on the extraction method. The yield of chitosan extracted from green mussel shells in the present study was lower than the yield of chitosan from the shell of a green mussel shell (11.60%) [2], cicada slough (28.2%), silkworm chrysalis (3.1%), mealworm (2.5%), grasshopper (5.7%), shrimp shells (14.5%) [5], mantis shrimp shells (14.13–15.79%) [11], horse mussel (*Modiolus modiolus*) (10.21%) [14], and house cricket (*Brachytrupes portentosus*) (2.4–5.8%) [20]. Green mussel shells can be used to extract chitosan, which is a potential starting material for expanding the chitosan source.

The degree of deacetylation (DDA) is an important parameter for determining the quality of chitosan. The higher the purity of the chitosan, the higher the DDA. This parameter also directly affects the biological, functional, and physicochemical properties of the obtained chitosan [9]. Furthermore, the DDA is used to indicate the effectiveness of the chemical deacetylation process for removing acetyl groups. The DDA values of chitosan obtained from small green mussel shells (CHS) and big green mussel shells (CHB) were calculated from FTIR studies and were 32.71% and 52.56%, respectively (Table 2). The commercial chitosan (CH) was analyzed as a reference sample with 70.42% DDA (Table 2). A significant difference in DDA was observed between CHS and CHB (*p* < 0.05). Furthermore, the low DDA may suggest an incomplete deacetylation process [7]. A similar DDA value has been reported on green mussel shell (48.68%) [2], mealworm cuticles (53.9%) [9], horse mussel (57.43%) [11], and *Pinna bicolor* shell (59.76%) [13]. However, the DDA of chitosan extracted from green mussel shells in the present study showed a lower value than that of crab shell (88.29%) [2], *P. visidis* (93.4%) [3], cicada slough (84.1%), silkworm chrysalis (85.5%), mealworm (85.9%), grasshopper (89.7%), and shrimp shells (91.2%) [5]. The differences in the results of DDA might be attributed to the variation in species, nature, location, the initial compositions of raw material, chitosan processing methods (such as deproteinization, demineralization, decolorization, and deacetylation), and their conditions (heating temperature and time, and alkali concentrations) [7,8]. In addition, further study is needed to improve the effectiveness of the extraction method, such as extending the heating time and/or increasing the alkali concentration, to obtain chitosan that can be comparable to commercial chitosan and can be widely used in many industries. In recent years, the most challenging part of chitosan extraction has been obtaining chitosan materials with high purity and good properties.

#### 3.2.3. Solubility

One of the key parameters for determining the quality of chitosan is its solubility. Chitosan with higher purity and quality has higher solubility [8]. The solubility of the chitosan prepared from different sizes of green mussel shells (CHS and CHB) in comparison with commercial chitosan (CH) is depicted in Table 2. The solubility values of CHS and CHB were 81.30% and 90.00%, while CH was completely soluble. Among the extracted chitosan, CHB was a bit more soluble than CHS (*p* < 0.05). In this study, the extracted chitosan had a lower solubility value than CH due to its lower DDA. This is related to the DDA of CH, which had a higher DDA than the extracted chitosan. However, the solubility of the obtained chitosan in this study was higher than that of chitosan extracted from other sources, such as shrimp shell (70%), fish scale (78%), and crab shell (60%) [7]. According to Kumari et al. [7], there are several factors that can affect the solubility of chitosan, such as the deacetylation process (heating temperature, reaction time, and alkali concentration), the source of starting materials, particle size, and the solid-to-solvent ratio.

#### 3.2.4. Water-Binding Capacity and Fat-Binding Capacity

The water-binding capacity (WBC) of the chitosan prepared from different sizes of green mussel shells (CHS and CHB) in comparison with commercial chitosan (CH) is depicted in Table 2. The WBC values of CHS, CHB, and CH were 187.97%, 209.55%, and 466.73%, respectively. The WBC values of both forms of extracted chitosan were found to be lower than that of CH (*p* < 0.05). However, no significant difference in WBC between CHS and CHB was observed (*p* > 0.05). According to Kumari et al. [25], the higher DDA provides more −NH_2_ groups to attract water and reduce crystallinity, resulting in increased penetration of water molecules. Kumari et al. [7,25] reported the WBC values for chitosan from fish scales, shrimp shells, and crab shells were 492%, 358%, and 138%, respectively. The sequence of extraction processes, such as deproteinization and demineralization, also had a pronounced effect on the WBC of the obtained chitosan [26].

The fat-binding capacity (FBC) of the chitosan prepared from different sizes of green mussel shells (CHS and CHB) in comparison with CH is depicted in Table 2. The FBC of extracted chitosan and CH was measured using soybean oil. The FBC values of CHS, CHB, and CH were 249.02%, 289.23%, and 452.26%, respectively. Luo et al. [5] and Kumari et al. [7] reported a similar outcome for chitosan extracted from shrimp shells (246%), fish scales (226%), and grasshoppers (275%). However, the FBC values of both forms of extracted chitosan in this study were lower than those of chitosan from mealworm (574%), silkworm chrysalis (412%) [5], black tiger shrimp shells (397–428%) [17], and giant freshwater prawn (372.33%) [27]. The FBC value generally depends on the source of raw material and chitosan production [25]. Some studies reported that the FBC of extracted chitosan was affected by the order of extraction processing [25,26]. The results on WBC and FBC are useful for the application of chitosan in various industries, such as food, cosmetics, and water treatment.

#### 3.2.5. FTIR Analysis

**Figure 2:**
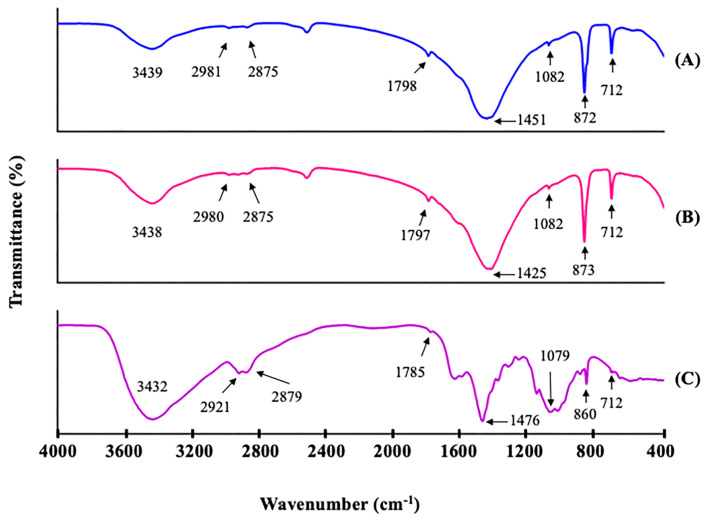
FTIR spectra of (**A**) chitosan extracted from small size shells (CHS), (**B**) chitosan extracted from big size shells (CHB), and (**C**) commercial chitosan (CH).

The FTIR spectra of the chitosan prepared from different sizes of green mussel shells (CHS and CHB) in comparison with commercial chitosan (CH) are presented in Figure 2. The CHS and CHB exhibited a broad band at 3439.26 and 3438.44 cm^−1^, respectively, while the CH showed a band at 3432.41 cm^−1^, which corresponds to the O–H and N–H stretching vibrations, as well as inter- and intramolecular hydrogen bonding [12]. The peaks at 2981.13 and 2875.49 cm^−1^ for CHS, 2980.87 and 2875.89 cm^−1^ for CHB, and 2921.63 and 2879.50 cm^−1^ for CH, were assigned to C–H asymmetric and symmetric stretching vibration, respectively [12]. The bands around 1798.40 cm^−1^ (CHS), 1797.57 cm^−1^ (CHB), and 1785.20 cm^−1^ (CH) represent the the C=O stretching vibration of the amide I [16]. The peaks at 1503.41 and 1451.41 cm^−1^ for CHS, 1504.12 and 1425.90 cm^−1^ for CHB, and 1599.32 and 1476.24 cm^−1^ for CH were assigned to N–H bending of amide (amide-II) and CH_2_ bending, respectively [20]. The saccharide band around wavenumbers of 1169.62, 1161.06, and 1154.68 cm^−1^ for CHS, CHB, and CH, respectively, was due to antisymmetric stretching of the C–O–C bridge. The peak representing C–O stretching vibration in alcohol was observed at 1082.73 cm^−1^ (CHS), 1082.41 cm^−1^ (CHB), and 1079.66 cm^−1^ (CH). The peaks at 872.90 cm^−1^ (CHS), 873.33 cm^−1^ (CHB), and 860.54 cm^−1^ (CH) were ascribed to the C-H out-of-plane vibration of the ring of monosaccharides. The peaks at 712.50 and 587.51 cm^−1^ (CHS), 712.50 and 587.88 cm^−1^ (CHB), and 712.52 and 525.52 cm^−1^ (CH) were assigned to out-of-plane bending NH and out-of-plane bending CO [14]. In addition, both CHS and CHB showed FTIR spectra patterns corresponding to the characteristics of chitosan and similar to those of CH. Furthermore, the FTIR spectra of both CHS and CHB were similar to those of chitosan from house cricket, horse mussel, and *Pinna bicolor* pen shell [13,14,20].

#### 3.2.6. XRD Analysis

**Figure 3:**
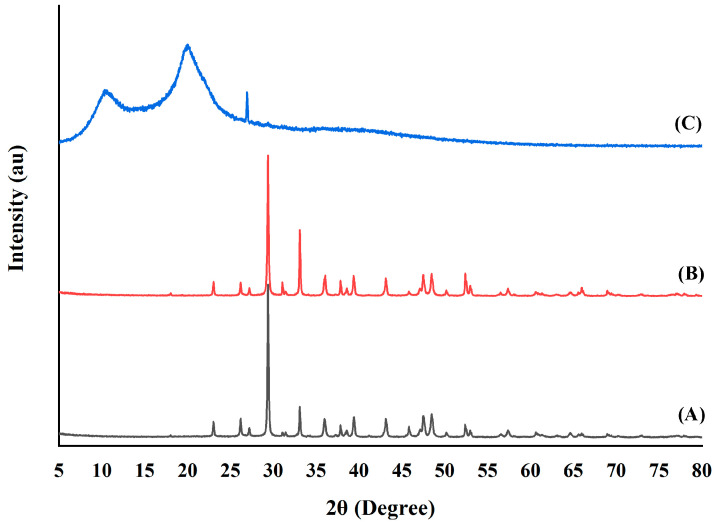
X-ray diffraction patterns of (**A**) chitosan extracted from small size green mussel shells, (**B**) chitosan extracted from big size green mussel shells, and (**C**) commercial chitosan.

Generally, polymorphic substances with various crystalline structures are analyzed using X-ray diffraction. The X-ray diffraction patterns of the chitosan extracted from both sizes of green mussel shells (CHS and CHB) in comparison with commercial chitosan (CH) are depicted in Figure 3. In the XRD spectra of CHS, a total of 13 different peaks were observed at the 2θ values of 23°, 26°, 27°, 29°, 33°, 35°, 37°, 39°, 43°, 47°, 48°, 52°, and 57°. The CHB showed a total of 15 peaks at 23°, 26°, 27°, 29°, 31°, 33°, 36°, 37°, 39°, 43°, 47°, 48°, 52°, 57°, and 65°. Both CHS and CHB showed two sharp peaks at 29° and 33° and intense peaks at 33–39°, which denote the presence of the hydroxyapatite mineral substance [25]. Similar observations were made by Kumari et al. [7,25] and Sebastian et al. [28]. The CH had crystalline reflections at 10°, 20°, 27°, and 29°. The CH showed a very sharp peak at 10° and 20°, whereas both CHS and CHB showed minor peak at 18°. The diffraction patterns of both CHS and CHB were different from those of CH due to the presence of some minerals and a difference in the origin of starting material. From this analysis, the XRD patterns of chitosan extracted from green mussel shells match closely with those of chitosan extracted from crab shell as reported by Kumari et al. [7] and chitosan extracted from *Pinna bicolor* pen shell as reported by Sudatta et al. [13].

### 3.3. Characterization of Chitosan Blended Films

#### 3.3.1. Film Thickness and Mechanical Properties

Thickness is one parameter that affects the other properties of films, such as mechanical properties, water vapor permeability, and optical properties (light transmission and transparency). The thickness of the commercial chitosan (CH)/extracted chitosan (chitosan extracted from small shells, CHS, or chitosan extracted from big shells, CHB) blended films is presented in Table 3. The thickness values of CH/CHS blended film and CH/CHB blended film were 0.034 mm, while that of the CH film was 0.028 mm. Significant differences were observed in the CH film and the CH/extracted chitosan blended films (*p* < 0.05). The addition of extracted chitosan could increase the thickness of the CH film, regardless of the type of extracted chitosan. The increased solid content in the CH film may be the cause of the blended films’ thicker films. Similar trends were presented for gelatin/gum films reinforced with chitosan nanoparticles [29] and chitosan/gelatin/modified chitosan-silver nanoparticles blended films [30].

The mechanical properties of biopolymer films are related to their ability to resist mechanical or physical damage to protect packaged products. Tensile strength (TS) and elongation at break (EAB) of CH film were 37.74 MPa and 0.26%, respectively (Table 3). The addition of extracted chitosan from both sizes of shells in the CH film increased the TS (41.81–45.97 MPa) and showed a significant difference when compared with the CH film (*p* < 0.05). This indicates that the addition of extracted chitosan could enhance the film’s strength. The extracted chitosan may induce hydrogen bonding between the CH/CHS or CH/CHB polymeric matrix during the formation of CH/extracted chitosan blended films, resulting in increased TS. The EAB generally had a contrast with TS: TS increased with decreasing EAB. The EAB of the CH film was 0.26%, while those of the CH/extracted chitosan films were 0.23–0.25% (Table 3). No significant difference was observed in EAB between the CH film and the CH/CHS blended film (*p* < 0.05). According to Benbettaïeb et al. [31], the mechanical properties of chitosan or chitosan blended films are dependent on a number of factors, including the DDA level of the chitosan, the polymer molecular mass, the solubilization method, the pH of the FFS, and the drying condition. The compactness of CH/extracted chitosan (CHS or CHB) polymeric films resulted in enhanced mechanical properties.

#### 3.3.2. Visual Appearance and Color Analysis of Chitosan Blended Films

**Figure 4:**
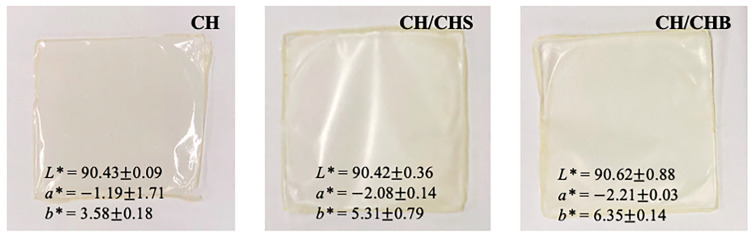
Visual aspect and color analysis of commercial chitosan film (CH), CH blended with chitosan extracted from small size shells film (CH/CHS), and CH blended with chitosan extracted from big size shells film (CH/CHB).

The visual appearance and color analysis of the CH/extracted chitosan blended films are depicted in Figure 4. All the film samples had a homogenous and smooth surface. Visually, all the films were transparent, as found by naked eye observation. However, the film samples became rigid after drying, which related to the TS result. The CH film had a clear and light-yellow color, while the CH/CHS and the CH/CHB blended films showed an opaque and yellow color. The L* and a* values of the CH film and the chitosan blended films seemed to have no significant difference (*p* > 0.05). However, the addition of extracted chitosan to the CH film affected the b* value by increasing the yellowness of the films (*p* < 0.05). This result is related to the result of the color attributes of chitosan powder as discussed above (related to the presence of β-1,4 linked 2-amino-2-deoxy-d-glucopyranose repeat units) [24]. Benbettaïeb et al. [31] also found that the chitosan film had a higher b* value when compared to gelatin film. According to these findings, the CH/extracted chitosan blended films can be used as see-through (transparent) packaging when the blended films are considered as a packaging material.

#### 3.3.3. Water Vapor Permeability (WVP)

The WVP values of the CH/extracted chitosan (CHS or CHB) blended films are presented in Table 3. The CH/CHS blended film exhibited the highest WVP value (2.30 × 10^−7^ g m m^−2^ s^−1^ Pa^−1^), followed by the CH/CHS blended film (1.96 × 10^−7^ g m m^−2^ s^−1^ Pa^−1^) and the CH film (1.87 × 10^−7^ g m m^−2^ s^−1^ Pa^−1^), respectively (*p* < 0.05). The addition of extracted chitosan could increase the moisture absorption of blended films due to increased thickness. The WVP of the blended films increased with thickness, suggesting that the blended film has a water affinity that could be described by the hygroscopic nature of the chitosan [31]. A similar observation has been reported for mantis shrimp chitosan/agarose composite films [11]. WVP should be generally as low as possible in order to avoid the permeability of water vapor from the outside to the inside of packaged foods. However, the selected WVP value of the developed biopolymer films depends on the specification of the product and its intended use.

#### 3.3.4. Fourier Transform Infrared (FTIR) Analysis

**Figure 5:**
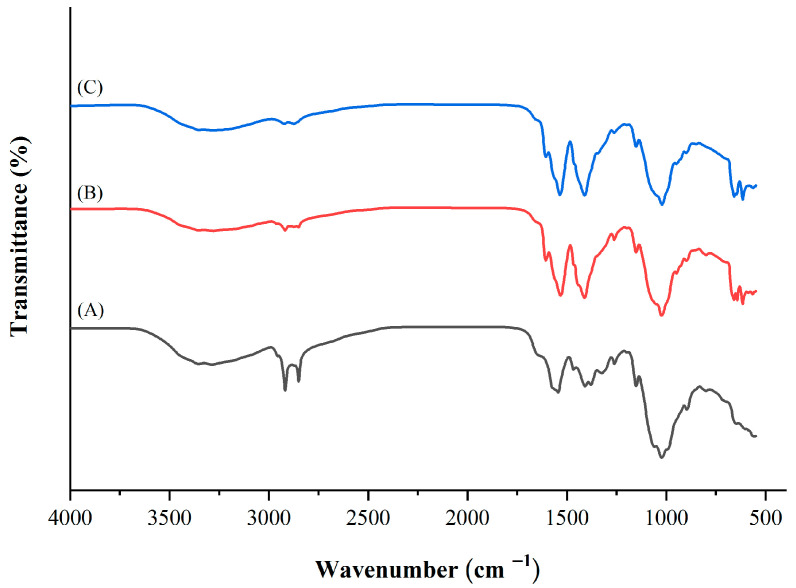
FTIR spectra of (**A**) CH film, (**B**) CH/CHS blended film, and (**C**) CH/CHB blended film.

The FTIR spectra of the CH/extracted chitosan blended films are depicted in Figure 5. The position of relevant peaks in the FTIR spectra of CH film was similar to those described by Yarnpakdee et al. [11], Koc et al. [15], and Benbettaïeb et al. [31]. The broad band of CH films at 3353.56 and 3273.42 cm^−1^ corresponded to N–H and O–H stretching vibrations, as well as inter- and intramolecular hydrogen bonding [12]. The characteristic peaks were at 2917.93 and 2850.12 cm^−1^ (C–H asymmetric and symmetric stretching vibration), 1606.60 cm^−1^ (C=O stretching; amide I), 1543.22 cm^−1^ (N–H bending; amide II), and 1407.59 cm^−1^ (CH_2_ bending) [15]. The broad peak at wavenumber 1023.33 cm^−1^ indicated C–O stretching vibration of chitosan [15]. The spectra of the CH/CHS and the CH/CHB blended films exhibited the characteristic peaks of CH. However, changes in wavenumber for –OH bond peak and amide II of both blended films were observed. The wavenumber of –OH bond peaks shifted from 3273.42 cm^−1^ for the CH film (without addition of CHS or CHB) to 3277.37 and 3282.90 cm^−1^ for the CH/CHS and the CH/CHS blended films, respectively. The –OH bond peak of the blended films shifted to a higher wavenumber due to the greatest formation of hydrogen bonding. For the amide II peak, the wavenumber was shifted to a lower wavenumber from 1543.22 cm^−1^ (without addition of CHS or CHB) to 1531.44 and 1535.75 cm^−1^ for the CH/CHS and the CH/CHS blended films, respectively. This was due to a hydrogen bonding interaction between CH and CHS or CHB forming in the film matrix. The results confirmed that the combination of CH and CHS or CHB could strengthen the interation between molecules via the hydrogen bond, resulting in the compatibility of these polymers in the blended films. This was consistent with the greater TS, which is shown in Table 3.

## 4. Conclusions

The extracted chitosan was successfully prepared from different sizes of green mussel shells. Both sizes of green mussel shells had a high content of ash and calcium carbonate. The different sizes of green mussel shells significantly affected the physicochemical properties of the obtained chitosan. The lower solubility of CHS and CHB was correlated with a lower DDA. The CHS and CHB showed poor physicochemical properties, especially in DDA, when compared with the CH. The XRD patterns demonstrated that both extracted chitosan samples comprised mineral traces. Furthermore, the addition of extracted chitosan (CHS or CHB) as a composite material to the CH film showed significant effects on the properties of the CH film by increasing the thickness value, film strength, WVP, and yellowness of the CH films. Further study is needed to improve the chitosan extraction process, which can enhance the physicochemical properties of the obtained chitosan.

## Figures and Tables

**Table 1:** Characterization of green mussel shells.

Composition	Small Shells (≤5.00 cm in Length)	Big Shells (>5.01 cm in Length)
Moisture (%)	0.79	0.91
Ash (%)	42.63	92.90
Fat (%)	0.03	0.03
Protein (%)	5.13	4.51
Calcium carbonate (%)	78.91	78.13

**Table 2:** Physicochemical properties of chitosan extracted from different sizes of green mussel shells in comparison with commercial chitosan.

Parameters	CHS	CHB	CH
Yield (%)	0.225 ± 0.022 ^a^	0.079 ± 0.019 ^b^	-
Degree of deacetylation (%, DDA)	32.71 ± 1.93 ^c^	52.56 ± 1.27 ^b^	70.42 ± 1.95 ^a^
Solubility (%)	81.30 ± 2.58 ^c^	90.00 ± 0.58 ^b^	100 ± 00 ^a^
Water-binding capacity (%, WBC)	187.97 ± 3.26 ^b^	209.55 ± 1.53 ^b^	466.73 ± 3.49 ^a^
Fat-binding capacity (%, FBC)	249.02 ± 2.49 ^c^	289.23 ± 2.35 ^b^	452.26 ± 2.99 ^a^
Color attributes - L* - a* - b*	90.51 ± 0.42 ^a^ 1.58 ± 0.05 ^a^ 5.03 ± 0.12 ^c^	90.42 ± 0.19 ^a^ 1.12 ± 0.05 ^b^ 8.48 ± 0.37 ^b^	86.12 ± 0.07 ^b^ 0.13 ± 0.03 ^c^ 19.68 ± 0.32 ^a^

Values are given as mean ± SD from *n* = 3 determination of yield, DDA, solubility, WBC, FBC; *n* = 5 determination of color attributes. Different superscripts in the same row indicate significant differences (*p* < 0.05).

**Table 3:** Thickness, mechanical properties, and water vapor permeability of the CH/extracted chitosan (CHS or CHB) blended films.

Properties	CH Film	CH/CHS Film	CH/CHB Film
Thickness (mm)	0.028 ± 0.0025 ^b^	0.034 ± 0.0020 ^a^	0.034 ± 0.0016 ^a^
Tensile strength (MPa)	37.74 ± 0.35 ^c^	41.81 ± 0.86 ^b^	45.97 ± 2.34 ^a^
Elongation at break (%)	0.26 ± 0.010 ^a^	0.25 ± 0.003 ^a^	0.23 ± 0.004 ^b^
Water vapor permeability (×10^−7^ g m m^−2^ s^−1^ Pa^−1^)	1.87 ± 0.16 ^b^	2.30 ± 0.19 ^a^	1.96 ± 0.20 ^ab^

Values are given as mean ± SD from *n* = 10 determination of thickness; *n* = 5 determination of mechanical properties; *n* = 3 determination of water vapor permeability. Different superscripts in the same row indicate significant differences (*p* < 0.05). CH film: commercial chitosan film, CH/CHS film: commercial chitosan blended with chitosan extracted from small size shells film, CH/CHB film: commercial chitosan blended with chitosan extracted from big size shells film.

## Data Availability

The data presented in this study are available on request from the corresponding author.
